# Anatomical and Three-Dimensional Study of the Female Feline Abdominal and Pelvic Vascular System Using Dissections, Computed Tomography Angiography and Magnetic Resonance Angiography

**DOI:** 10.3390/vetsci10120704

**Published:** 2023-12-14

**Authors:** Daniel Rojo Ríos, Gregorio Ramírez Zarzosa, Marta Soler Laguía, David Kilroy, Francisco Martínez Gomariz, Cayetano Sánchez Collado, Francisco Gil Cano, María I. García García, María Dolores Ayala Florenciano, Alberto Arencibia Espinosa

**Affiliations:** 1 Department of Anatomy and Comparative Pathological Anatomy, Veterinary Faculty, Campus de Espinardo, University of Murcia, 30100 Murcia, Spain; 2 Department of Animal Medicine and Surgery, Veterinary Faculty, Campus de Espinardo, University of Murcia, 30100 Murcia, Spain; 3 Veterinary Science Centre, University College Dublin, Belfield, D04 V1W8 Dublin, Ireland; 4 Support Research Service SACE-ACTI, University of Murcia, 30100 Murcia, Spain; 5 Department of Morphology, Veterinary Faculty, University of Las Palmas de Gran Canaria, 35413 Las Palmas, Spain

**Keywords:** computed tomography angiography, volume rendering, 3D printing, magnetic resonance angiography, TOF, abdominal vascular imaging, pelvic vascular imaging, anatomy, feline

## Abstract

**Simple Summary:**

The aim of this report was to depict the normal anatomy of the vascular structures of the abdominal and pelvic regions in two female mature cats using computed tomography angiography, magnetic resonance angiography and three-dimensional printing. Three feline cadavers were used for anatomical dissections. The cats were scanned after iodinated contrast media was injected, and three-dimensional computed tomography angiography images were obtained with different computer software such as RadiAnt 2023.1, Amira 5.6 and OsiriX MD 13.0.2. A magnetic resonance angiography study was made through a non-contrast enhanced time-of-flight sequence. We also performed three-dimensional print and gross dissections to aid the identification of the main vascular structures of these anatomical regions and allow comparisons with computed tomography angiography and magnetic resonance angiography images. The computed tomography angiography and magnetic resonance angiography provided good detail of the main abdominal and pelvic arteries and veins. Results of the current research may be used for other anatomical studies and in the assessment of several disorders of these regions.

**Abstract:**

This study describes the anatomical characteristics of the abdominal and pelvic vascular system of two healthy mature female cats via three-dimensional contrast enhanced computed tomography angiography, non-contrast enhanced magnetic resonance angiography and three-dimensional printing. Volume-rendering computed tomography angiography images were acquired from the ventral aspect using RadiAnt, Amira and OsiriX MD Dicom three-dimensional formats, and three-dimensional printing was obtained and compared with the corresponding computed tomography angiography images. Non-contrast enhanced magnetic resonance angiography was made using the time-of-flight imaging in ventral, oblique and lateral views. In addition, three cadavers with colored latex injection were dissected to facilitate the identification of the vascular structures. Three-dimensional computed tomography angiography showed the main vascular structures, whereas with the time-of-flight blood appeared with a high signal intensity compared with associated abdominal and pelvic tissues. Three-dimensional computed tomography angiography images and time-of-flight sequences provided adequate anatomical details of the main arteries and veins that could be used for future feline anatomical and clinical vascular studies of the abdomen and pelvis.

## 1. Introduction

In humans, helical computed tomography angiography (CTA) and magnetic resonance angiography (MRA) are powerful advanced imaging techniques for the clinical evaluation of the abdominal and pelvic vascular system [1,2]. The main advantages of these advanced image-based diagnostic methods are their speed in obtaining images with improved anatomic definition, better contrast resolution of vascular segments and the reduction of motion artifacts [1,3]. When compared with conventional angiography and ultrasonography, CTA and MRA avoid the superimposition of structures, provide superior contrast resolution, allow the production of multiplanar imaging and are less limited by operator experience [3,4,5].

In addition, the employment of iodinated contrast medium in CTA, or gadolinium in MRA improves the resolution of the vascular system and increases the contrast enhancement of parenchymal organs [1,5,6,7,8]. Non-contrast enhanced MR angiographic techniques also enable assessment of the abdominopelvic vasculature without administration of gadolinium-based contrast media [9]. These techniques include black-blood spin-echo and bright-blood gradient-echo pulse sequences imaging depending on the signal intensity of the vascular lumen [10,11]. CTA has the disadvantages of the use of high doses of ionizing radiation in patients and causing kidney disorders [5,8], whereas non-contrast magnetic resonance angiography can be applied for accurate diagnosis of vascular disorders without presenting those effects [12,13]. However, it should be mentioned that the availability of MRI devices capable of producing the TOF sequence is limited compared to the accessibility that both clinical veterinarians and researchers have to CT devices, which is why the latter is the first imaging option today.

By using different software, both modalities (CTA and MRA) quickly obtain volumetric information, which can later be analyzed slab by slab or by advanced volume reconstruction procedures [14,15] which permit the exploration of anatomical detail that would be difficult to evaluate using axial reconstructions alone [16]. Volume rendering (VR) is a technique for generating reconstruction images that outline the anatomical characteristics of the organs and associated blood vessels [5,6,17,18]. The Volume Rendering-3D reconstruction software varies from one CT scanner manufacturer to another, but they all work similarly on the CT workstation [16]. Likewise, there are several DICOM image viewer options available to analyze CT studies from the computer without using the CT workstation. To perform this study, we chose three different viewers that are normally used in the different veterinary fields: AMIRA in the case of research centers, OsiriX in the case of certified radiologists and RadiAnt in the case of clinical veterinarians (general practitioners). TOF techniques produce a difference between blood flowing and tissues and organs by handling of the value of the magnetization. This can be achieved by imaging planes selected and directed in a perpendicular orientation to blood circulation. With TOF methods, 2D or 3D acquisitions can be obtained. The employment of a flow-compensated gradient-echo MRI sequence from multiple thin imaging slabs from the vessels of interest generated 2D acquisitions [10,11,12]. Using a reconstruction technique results in a three-dimensional image of the vessels similar to conventional angiography [13,14].

In feline medicine, radiography and ultrasound have been the main diagnostic imaging techniques used for the abdomen and pelvis [19,20]. However, the use of CTA and MRA in cats have made it possible to obtain better resolution of the circulatory system. The major disadvantages of CTA and MRA include cost, limited equipment availability and the need for general anesthesia. In cats, anatomical studies using helical CTA have been limited to the evaluation of maxillary artery blood flow with the mouth closed and opened [21], the cardiac chambers and walls [22], the coronary arteries [23], the stomach and small intestine [24,25], the liver [24,25,26,27,28], the pancreas [24,25,29] and the kidneys [24,25,30,31,32].

Information about MRA in cats is limited to a few anatomical reports on the thorax [33,34] and the visualization of the abdominal aorta and external iliac arteries [35]. In veterinary medicine, 3D printings have also been used as resources to improve anatomical and physiological studies [25,28,36,37].

Our main objective was to describe the normal feline vascular structures of the abdominal and pelvic cavities using anatomical dissections with colored latex injection to compare it with CTA and MRA images, and also to use three different 3D VR software (OsiriX MD 13.0.2, Amira 5.6 and RadiAnt 2023.1) to decide if there are important differences between them, so as to be able to make recommendations depending on the accessibility of users to these programs.

## 2. Materials and Methods

### 2.1. Animals

Two healthy live female cats (*Felis silvestris catus*, L.) aged 2 and 6 years old and weighing 3.2 kg and 4.2 kg were used for the CTA and MRA, respectively. After clinical evaluation, the anesthetic protocols in both animals for each study consisted of premedication with a combination of medetomidine (Domtor, Orion Farma) 0.075 mg/kg and ketamine (Ketaset, Zoetis Inc., Kalamazoo, MI 100 mg/mL) 5 mg/kg injected IM, and induction with IV propofol (Propofol-Lipuro 1%, Braun) 4–6 mg/kg via the cephalic vein. The anesthesia was then maintained with a mixture of volatile anesthetic agents in oxygen (isoflurane (Forane, Abott) 2–3% in a flow of 0.8 L/min of O_2_). Mechanical ventilation was used throughout the studies at a rate of 10–12 breaths/min and the cats received IV physiological saline solution at 5 mL/h.

In the dissection study, three crossbreed female cat cadavers were used. The cats ranged in age from 2 to 4 years old, and each weighed approximately 4 kg, and were acquired from the Zoonoses Service of Murcia (Spain). The animals were humanely euthanized for reasons unrelated to this project. This study was supervised, and the research protocol authorized by the Animal Ethical Committee of Veterinary Medicine of the University of Murcia, Spain (REGA ES300305440012 CEEA: 305/2017; extended on 25/07/2022 as project Type II).

### 2.2. Anatomic Evaluation

Anatomical dissections allowed us to evaluate the quality of the images obtained with the chosen techniques (CT, MRI and 3D Printing) when identifying vascular structures. The fresh cat cadavers were transported to the dissection room and vascular pumping with 2% saline solution was carried out. These specimens were then injected via the common carotid artery and external jugular vein with red and blue latex (NV001, Ballons CP, Espinardo, Murcia, Spain), respectively. Subsequently, fresh cadavers with vascular repletion were frozen at −20 °C for at least 15 days to harden the latex before performing anatomical dissections. After the dissections, the cats were embalmed (1% formaldehyde, 5% glycerin, 10% isopropyl alcohol and 5% phenol and water) by immersion in a container. Several organs, and abdominal and pelvic vascular structures were labelled according to the Illustrated Veterinary Anatomical Nomenclature [38].

### 2.3. Computed Tomography Angiography and 3D Printing

Triple-phase contrast enhanced CT was performed obtaining the plain arterial phase (20 s after the start of injection of the contrast medium), the venous phase (40 s after the start of the injection), and the delayed phase (120 s after the start of the injection). Iomeprol (Iomeron, Madrid, Spain, 300 mgI/mL) was used as contrast medium and administered at a dose of 800 mgI/Kg, at 3 mL/s, via the cephalic vein with a power injector (Auto Enhance A-60; Nemoto-Kyorindo, Tokyo, Japan).

CTA was acquired with the animal in dorsal recumbency using a 16-slice CT unit (Toshiba Astelion, Toshiba Medical System, Madrid, Spain). The technical parameters were 120 kV tube voltage, 80 mAs tube current, 1.5 s tube rotation time, spiral pitch factor of 0.94 and 3 mm slice thickness. The display field of view was of 35 cm and the image matrix was 512 × 512. On the CT workstation, images were obtained with soft tissue and bone algorithms and reformatted in sagittal and dorsal planes, maximum intensity projection and VR. The images were reviewed in a PACS workstation using soft tissue (WW = 400, WL = 40) and bone windows (WW = 1500, WL = 300). Adjustments to image window width and level were made as needed.

To better evaluate the appearance of the vascular and pelvic structures, Dicom files of the venous phase were adjusted using three standard Dicom 3D software packages (OsiriX MD 13.0.2, Pixmeo, Bernex, Switzerland; AMIRA 5.6, Thermo Fisher Scientific, Waltham, MA, USA; RadiAnt DICOM viewer 2023.1; Medixant Co., Poznan, Poland) to produce three-dimensional volumetric representations. Subsequently, 3D printing of the arterial and venous systems of the abdominal and pelvic cavities was performed using the Slicer 3.0 program. Next, 3D printing was obtained using the Grabcad Print 1.71.7.21930 program (Waltham, MA, USA) and Stratasys F170-FDM printer (Los Angeles, CA, USA). Finally, the printed cast was painted with different colors to identify organs and vascular structures.

### 2.4. Magnetic Resonance Angiography

Non-contrast enhanced MRA was performed with a high-field (1.5 Tesla) MRI scanner (General Electric Sigma Excite, Schenectady, NA, USA), using a human head coil of 8-channel. The animal was placed in dorsal recumbency, and retrospective electrocardiographic gating (ECG) was used to mitigate motion artifacts in the images by the visceral motion and pulsatility of the small vessels. Using software tools of these standard packages, the viscera (small and large intestine, liver, spleen) located below the abdominal and pelvic roof were manually removed for a better visualization of the vascular structures.

Ventral, oblique and lateral 3D TOF images were acquired with the following parameters: TR: 25 ms; TE: 6.9 ms; Slice thickness: 2 mm; Flip angle: 20 and Acquisition Matrix: 256 × 256. Abdominal and pelvic vascular structures were assessed according to their hyperintensity signal and compared to the three-dimensional volume-rendering CT images, 3D printing and anatomical dissections. The interpretation of the CTA, 3D printing and MRA images was based on the study of anatomy textbooks [39,40,41] and gross dissections to facilitate the identification of the abdominal and pelvic vascular structures.

## 3. Results

### 3.1. Gross Dissections

**Figure 1:**
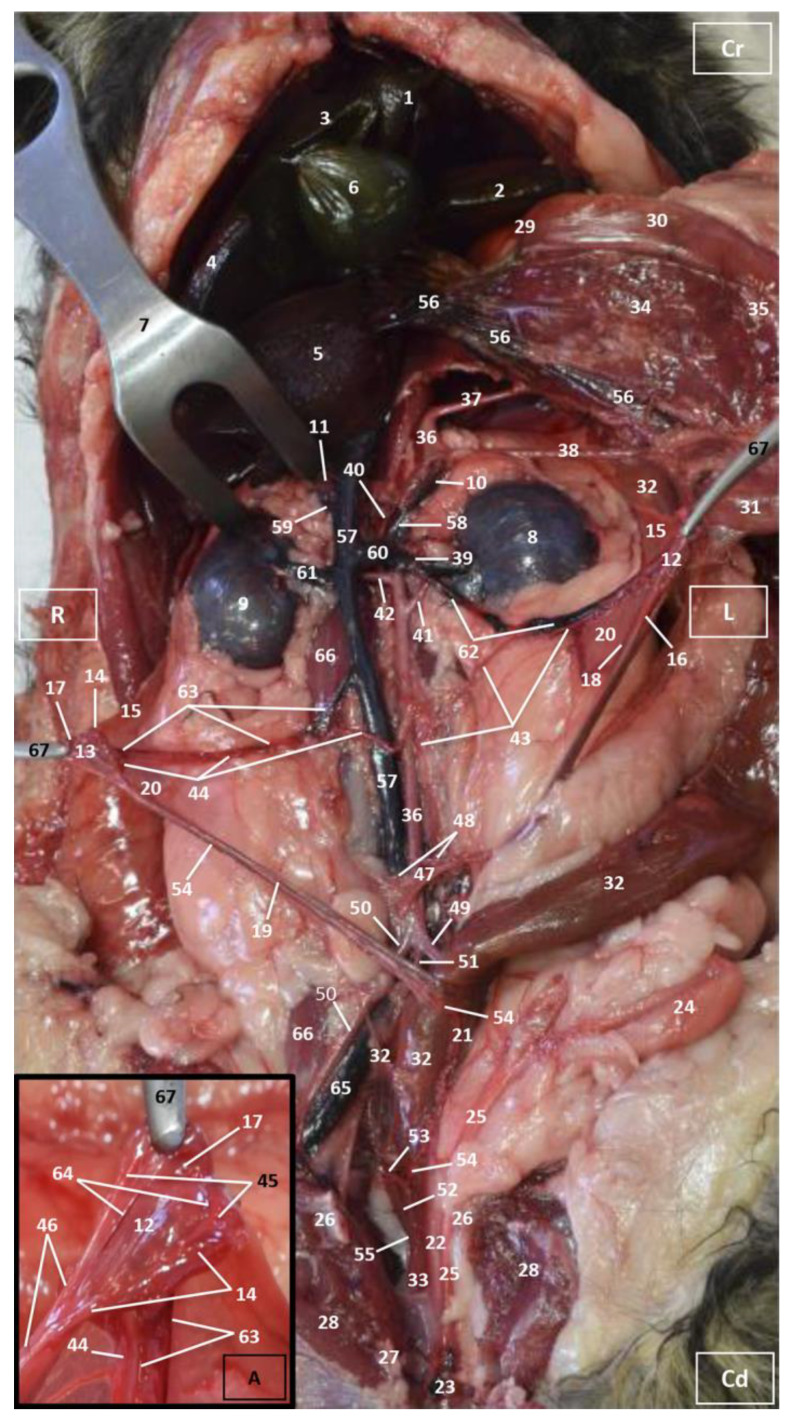
Deep anatomical dissection of the abdominal and pelvic cavities of the cat. Ventral view. The small and large intestine and right lobe of the pancreas has been displaced to the left. The right hepatic lobes have been displaced cranially using a surgical abdominal separator to observe the vascularization of the abdominal roof. The ovaries have been clamped from the mesovarium in order to clearly observe the irrigation of the right and left uterine horns. The arteries and veins have been injected with red and blue latex, respectively. (A) Detail of the ovarian irrigation. L = Left; R = Right; Cr = Cranial; Cd = Caudal. 1. Liver: left medial lobe; 2. Liver: left lateral lobe; 3. Liver: quadrate lobe; 4. Liver: right medial lobe; 5. Liver: right lateral lobe; 6. Gallbladder; 7. Abdominal surgery retractor; 8. Left kidney; 9. Right kidney; 10. Left adrenal gland; 11. Right adrenal gland; 12. Left ovary; 13. Right ovary; 14. Right uterine tube; 15. Mesovarium: suspensory ligament; 16. Mesovarium: proper ligament of the ovary; 17. Mesosalpinx; 18. Left uterine horn; 19. Right uterine horn; 20. Broad ligament: mesometrium; 21. Uterus: body; 22. Vagina; 23. Vagina: vestibule; 24. Urinary bladder; 25. Female urethra; 26. Pubis (sectioned); 27. Ischium (sectioned); 28. Musculature of the thigh (sectioned); 29. Duodenum: cranial part; 30. Duodenum: descending part; 31. Duodenum: ascending part; 32. Descending colon; 33. Rectum; 34. Pancreas: right lobe; 35. Mesoduodenum; 36. Abdominal aorta; 37. Celiac artery; 38. Cranial mesenteric artery; 39. Left caudal adrenal artery; 40. Right middle adrenal artery; 41. Left renal artery; 42. Right renal artery; 43. Left ovarian artery; 44. Right ovarian artery; 45. Right ovarian artery: tubal branch; 46. Right ovarian artery: uterine branch; 47. Caudal mesenteric artery; 48. Right and left deep circumflex iliac arteries; 49. Left external iliac artery; 50. Right external iliac artery; 51. Abdominal aorta: final stretch; 52. Right internal pudendal artery; 53. Right vaginal artery; 54. Right uterine artery; 55. Right middle rectal artery; 56. Portal vein; 57. Caudal vena cava; 58. Left adrenal vein; 59. Right adrenal vein; 60. Left renal vein; 61. Right renal vein; 62. Left ovarian vein; 63. Right ovarian vein; 64. Right ovarian vein: ovarian plexus; 65. Right common iliac vein; 66. Psoas major muscle; and 67. Hemostatic forceps.

**Figure 2:**
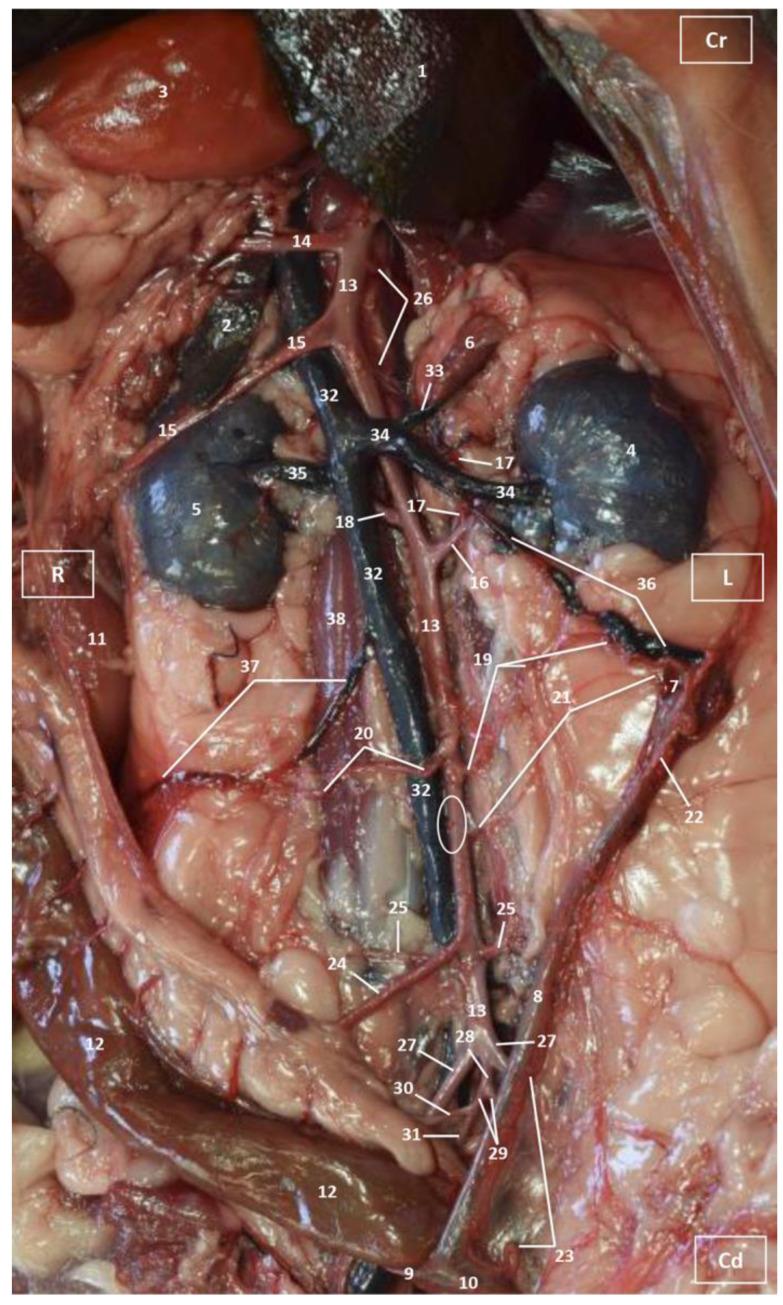
Deep anatomical dissection plane of the abdominal cavity of the cat. Ventral view. The small and large intestine have been displaced to the right. The arteries have been filled with red latex and the veins are blue. L = Left; R = Right; Cr = Cranial; Cd = Caudal. 1. Liver: left lateral lobe; 2. Liver: right lateral lobe; 3. Stomach; 4. Left kidney; 5. Right kidney; 6. Left adrenal gland; 7. Left ovary; 8. Left uterine horn; 9. Right uterine horn; 10. Uterus: body; 11. Duodenum: ascending portion; 12. Descending colon; 13. Abdominal aorta; 14. Celiac artery; 15. Cranial mesenteric artery; 16. Left renal artery; 17. Left caudal adrenal artery; 18. Right renal artery; 19. Left ovarian artery; 20. Right ovarian artery; 21. Left accessory ovarian artery; 22. Right ovarian artery: uterine branch; 23. Left uterine artery; 24. Caudal mesenteric artery; 25. Right and left deep circumflex iliac arteries; 26. Lumbar arteries; 27. Left and right external iliac arteries; 28. Abdominal aorta: final portion; 29. Left and right internal iliac arteries; 30. Caudal gluteal artery; 31. Right internal pudendal artery; 32. Caudal vena cava; 33. Left adrenal vein; 34. Left renal vein; 35. Right renal vein; 36. Left ovarian vein; 37. Right ovarian vein; 38. Psoas major muscle; and ellipse: a small sketch of the start of the obliterated right accessory ovarian artery.

**Figure 3:**
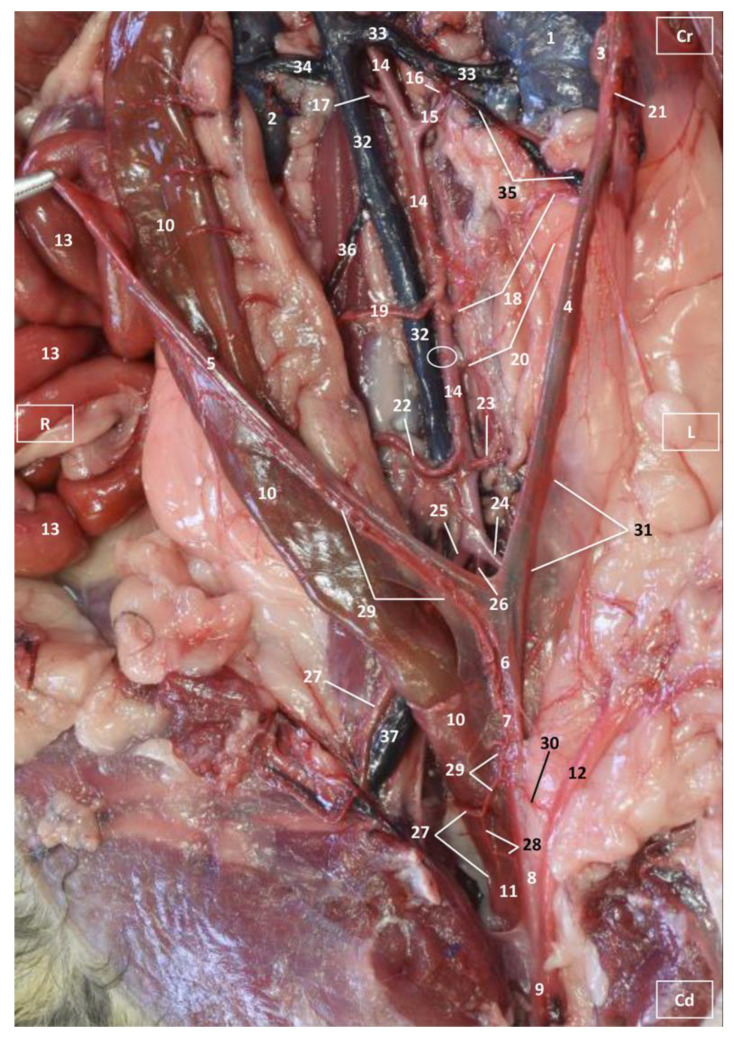
Deep anatomical dissection of the abdominal and pelvic cavities of the cat. Detail of the uterine irrigation. Left side view. The small and large intestine have been displaced to the right. The arteries have been filled with red latex and the veins are blue. L = Left; R = Right; Cr = Cranial; Cd = Caudal. 1. Left kidney; 2. right kidney; 3. left ovary; 4. left uterine horn; 5. right uterine horn; 6. uterus: body; 7. uterus: neck; 8. vagina; 9. vagina: vestibule; 10. descending colon; 11. rectum; 12. female urethra; 13. jejunum: loops; 14. abdominal aorta; 15. left renal artery; 16. left caudal adrenal artery; 17. right renal artery; 18. left ovarian artery; 19. right ovarian artery; 20. accessory ovarian artery; 21. right ovarian artery: uterine branch; 22. caudal mesenteric artery; 23. left deep circumflex iliac artery; 24. left external iliac artery; 25. right external iliac artery; 26. abdominal aorta: final stretch; 27. right middle rectal artery; 28. right vaginal artery; 29. right uterine artery; 30. urethral artery; 31. left uterine artery; 32. caudal vena cava; 33. left renal vein; 34. right renal vein; 35. left ovarian vein; 36. right ovarian vein; 37. common iliac vein; and ellipse: a small portion of the start of the obliterated right accessory ovarian artery.

**Figure 4:**
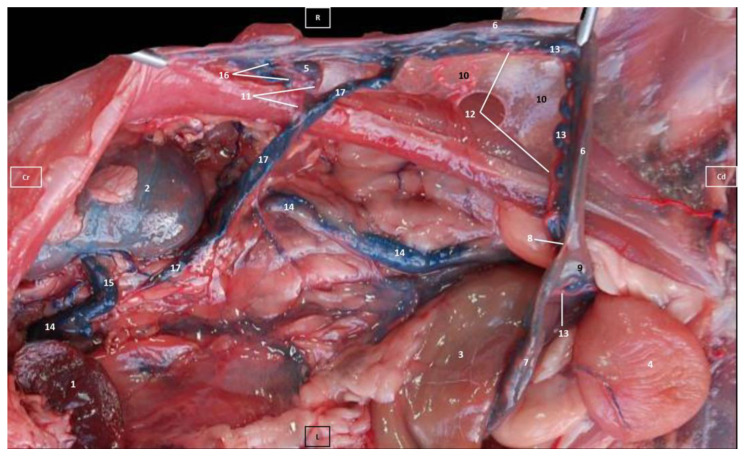
Deep dissection of the abdominal cavity of the cat. Left side view. The small and large intestine and left kidney have been displaced ventrally. The right uterine horn has been clamped dorsally with two hemostats to observe vascularization. The arteries have been filled with red latex and the veins with blue. The right ovarian vein drains into the caudal vena cava, L = left; R = right; Cr = cranial; Cd = caudal. 1. Spleen; 2. right kidney; 3. descending colon; 4. urinary bladder; 5. right ovary; 6. right uterine horn; 7. left uterine horn; 8. intercornual ligament; 9. uterus: body; 10. broad ligament: mesometrium; 11. right ovarian artery; 12. right uterine artery; 13. right uterine vein; 14. caudal vena cava; 15. right renal vein; 16. right ovarian vein: ovarian plexus; and 17. right ovarian vein.

Four figures corresponding to anatomical dissections with colored latex injection are presented. Figure 1, Figure 2 and Figure 3 were obtained from the abdominal and pelvic roof and Figure 4 from the abdominal lateral wall. Several abdominal and pelvic arteries and veins were well-visualized due to the colored latex substance injected.

#### 3.1.1. Arterial System

The abdominal aorta is located in the dorsal abdomen, and its first abdominal branch is the celiac artery that supplies the celiac organs (stomach, liver, spleen, pancreas, and proximal duodenum). Caudal to the celiac artery is the cranial mesenteric artery that branches through the small and large intestine. The caudal mesenteric artery supplies the terminal parts of the digestive system; all these branches were observed (Figure 1 and Figure 2). We differentiated caudal to the cranial mesenteric artery, the right middle and left caudal adrenal arteries that supply the adrenal glands (Figure 1 and Figure 2). Caudal to the cranial mesenteric artery (Figure 1), the right renal artery arises, passing dorsal to the caudal vena cava and the right renal vein until it reaches the renal hilus. The left renal artery emerges caudal to the right renal artery, close to the roof of the abdomen and dorsolateral to the left ovarian and renal veins. In addition, it can be seen that the left caudal adrenal artery originates from the left renal artery (Figure 2) and that the right middle adrenal artery is derived from the abdominal aorta, subsequently bifurcating into two branches that course towards the right adrenal gland (Figure 1).

To the right of the abdominal aorta and caudal to the left renal artery, the right ovarian artery branches off, coursing ventral to the caudal vena cava. It continues ventral to the psoas major muscle and, together with the right ovarian vein and supported by the broad ligament, they reach the right ovary. Slightly caudal to the right ovarian artery, the path of the left ovarian artery can be seen which, together with the left ovarian vein, reaches the left ovary (Figure 1, Figure 2 and Figure 4). In addition, we observed the uterine branch of the right ovarian artery, a large caliber vessel which supplies the right uterine horn and anastomoses with the right uterine artery. The tubal branch of the right ovarian artery is significantly smaller in caliber (Figure 1 and Figure 1A). Caudal to the ovarian arteries, we observed an accessory branch of the left ovarian artery coursing through the broad ligament (mesometrium) reaching the mesovarium and mesosalpinx, close to the ovary. We appreciated only a small portion of the origin of the obliterated right accessory ovarian artery, indicated with an ellipse (Figure 2 and Figure 3). Caudal to the ovarian arteries is the origin of the caudal mesenteric artery that supplies the distal transverse colon, the descending colon and the cranial portion of the rectum. We observed the termination of the abdominal aorta caudal to the origin of the deep circumflex iliac arteries. First, we observed the large caliber bifurcation of the external iliac arteries (right and left). Caudal to this, the aorta ends at the bifurcation of the internal iliac arteries (right and left) and the median sacral artery.

The internal iliac arteries divide into two branches, the caudal gluteal and the internal pudendal arteries (Figure 1, Figure 2 and Figure 3). The right internal pudendal artery that courses medially to the ischial spine was displaced to the left (Figure 1). It gives off the right vaginal artery, which in turn gives off the middle rectal artery. In addition, the right and left uterine arteries (very tortuous) were clearly seen as branches of the right (Figure 1 and Figure 3) and left vaginal arteries that travel lateroventrally to the rectum to reach the vagina. Before reaching the vagina, the right uterine artery branches off and courses cranioventrally to the uterus (Figure 1). The right and left uterine arteries (Figure 3) anastomose with the uterine branches of the right and left ovarian arteries, respectively. Finally, we identified the urethral artery arising from the uterine artery (Figure 3).

#### 3.1.2. Venous System

We observed how the caudal vena cava is located parallel to the abdominal aorta. Conveying venous blood from the pelvic limb and the pelvic cavity, we could appreciate the right common iliac vein entering the caudal vena cava. Arriving from the ovaries, the right ovarian vein flowed directly into the caudal vena cava, while the left ovarian vein drained into the left renal vein. We also identified the right adrenal vein draining into the caudal vena cava; however, the left adrenal vein emptied into the left renal vein. Both renal veins entered the caudal vena cava. In addition, we were able to observe the portal vein coursing towards the porta hepatis (Figure 1, Figure 2, Figure 3 and Figure 4). Finally, we saw the ovarian venous plexus filled with colored blue latex, as well as the uterine veins that appeared dilated and surrounded by the uterine artery (Figure 4).

### 3.2. Computed Tomography Angiography and 3D Printing

Three-dimensional volume-rendering images using RadiAnt, Amira and OsiriX DICOM viewers are presented. An additional image was obtained by 3D printing. Volume-rendering showed an adequate representation of several abdominal and pelvic arteries and veins, and these were identified by their elevated CT density by the administration of iodinated contrast media.

#### 3.2.1. Arterial System

**Figure 5:**
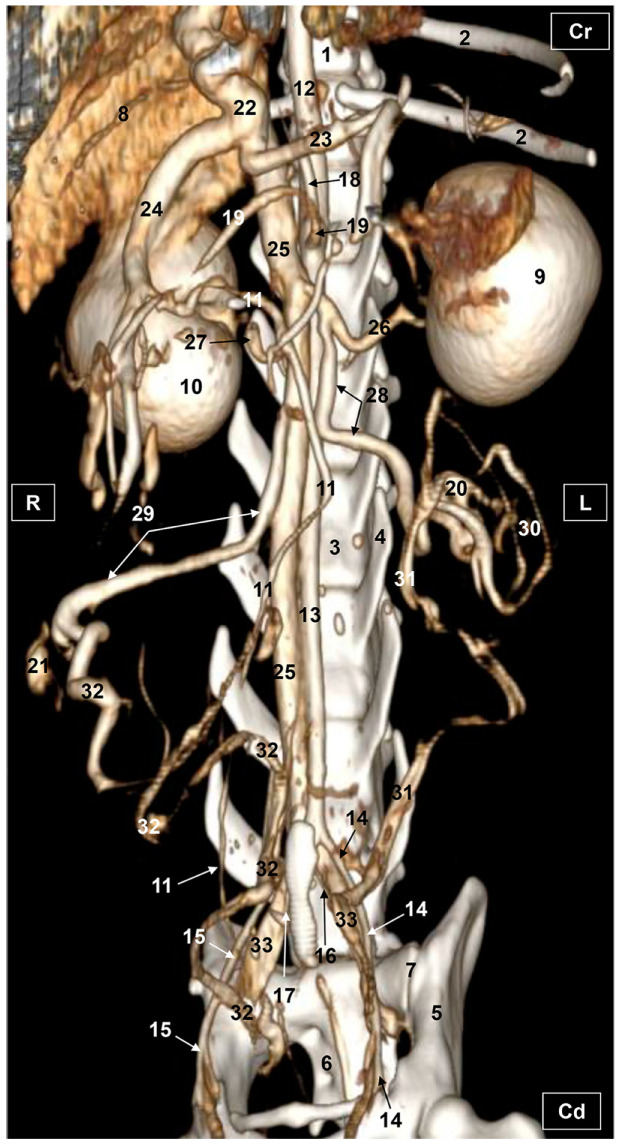
Three-dimensional reconstruction image of the abdominal and pelvic arterial and venous system in a female feline. Radiant VR. Ventral view. R = Right. L = Left. Cr = Cranial. Cd = Caudal. 1. Thoracic vertebra; 2. ribs; 3. lumbar vertebra; 4. costiform process; 5. ilium bone: wing; 6. sacrum: ventral face; 7. sacroiliac joint; 8. liver (sectioned); 9. left kidney; 10. right kidney; 11. right ureter; 12. descending aorta: thoracic aorta; 13. abdominal aorta; 14. left external iliac artery; 15. right external iliac artery; 16. left internal iliac artery; 17. right internal iliac artery; 18. celiac artery; 19. cranial mesenteric artery; 20. left ovary; 21. right ovary 22. portal vein; 23. splenic vein; 24. cranial mesenteric vein; 25. caudal vena cava; 26. left renal vein; 27. right renal vein; 28. left ovarian vein; 29. right ovarian vein; 30. ovarian vein: ovarian plexus; 31. left uterine artery and vein; 32. right uterine vein; and 33. left and right common iliac vein.

**Figure 6:**
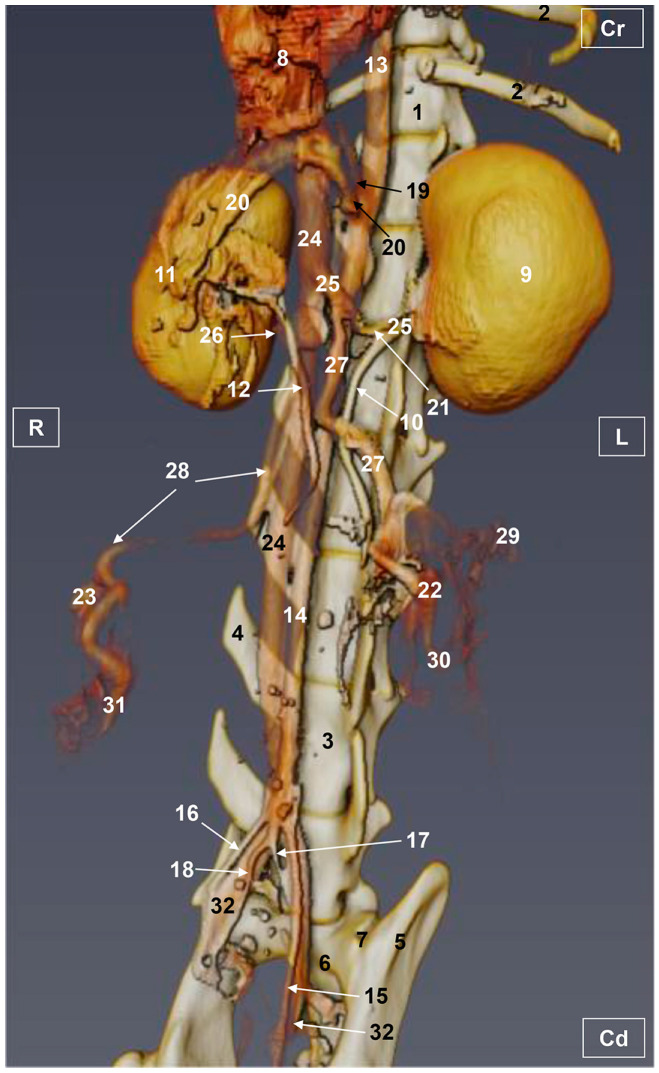
Three-dimensional reconstruction image of the abdominal and pelvic arterial and venous system in a female feline. Amira VR. Ventral view. R = Right. L = Left. Cr = Cranial. Cd = Caudal. 1. Thoracic vertebra; 2. ribs; 3. lumbar vertebra; 4. costiform process; 5. ilium bone: wing; 6. sacrum: ventral face; 7. sacroiliac joint; 8. liver (sectioned); 9. left kidney; 10. left ureter; 11. right kidney; 12. right ureter; 13. descending aorta: thoracic aorta; 14. abdominal aorta; 15. left external iliac artery; 16. right external iliac artery; 17. left internal iliac artery; 18. right internal iliac artery; 19. celiac artery; 20. cranial mesenteric artery; 21. left renal artery; 22. left ovary; 23. right ovary; 24. caudal vena cava; 25. left renal vein; 26. right renal vein; 27. left ovarian artery and vein; 28. right ovarian vein; 29. ovarian vein: ovarian plexus; 30. left uterine artery and vein; 31. right uterine vein; and 32. left and right common iliac vein.

**Figure 7:**
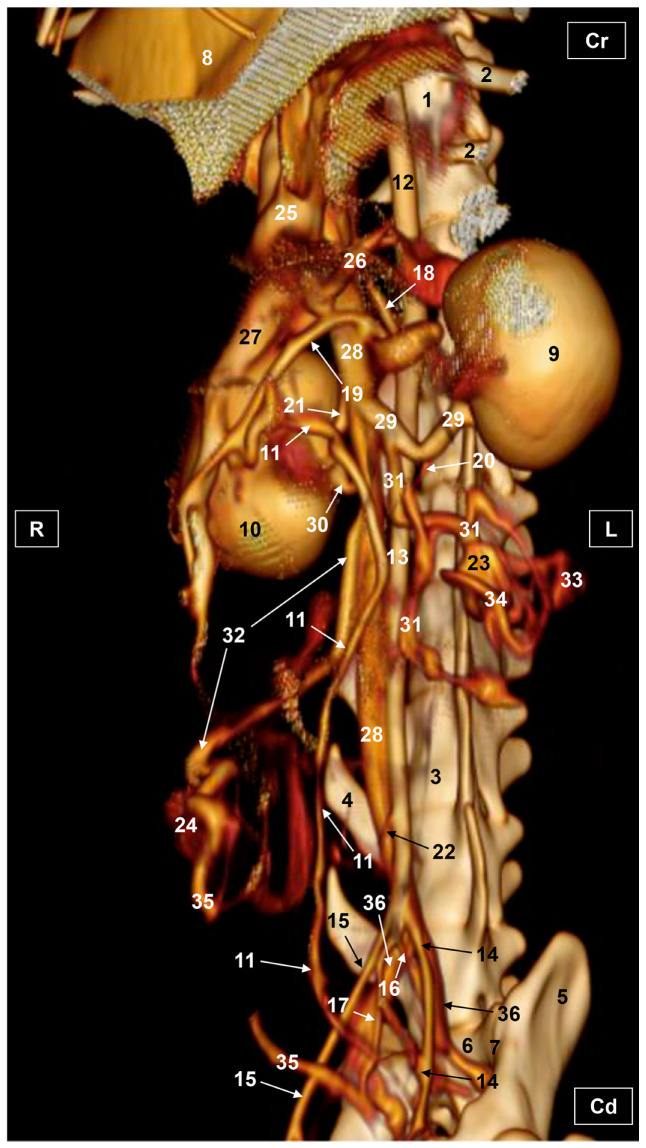
Three-dimensional reconstruction image of the abdominal and pelvic arterial and venous system in a female feline. OsiriX volumen rendering. Ventral view. R = Right. L = Left. Cr = Cranial. Cd = Caudal. 1. Thoracic vertebra; 2. ribs; 3. lumbar vertebra; 4. costiform process; 5. ilium bone: wing; 6. sacrum: ventral face; 7. sacroiliac joint; 8. liver (sectioned); 9. left kidney; 10. right kidney; 11. right ureter; 12. descending aorta: thoracic aorta; 13. abdominal aorta; 14. left external iliac artery; 15. right external iliac artery; 16. left internal iliac artery; 17. right internal iliac artery; 18. celiac artery; 19. cranial mesenteric artery; 20. left renal artery; 21. right renal artery; 22. caudal mesenteric artery; 23. left ovary; 24. right ovary 25. portal vein; 26. splenic vein; 27. cranial mesenteric vein; 28. caudal vena cava; 29. left renal vein; 30. right renal vein; 31. left ovarian vein; 32. right ovarian vein; 33. ovarian vein: ovarian plexus; 34. left uterine artery and vein; 35. right uterine artery and vein; and 36. left and right common iliac vein.

**Figure 8:**
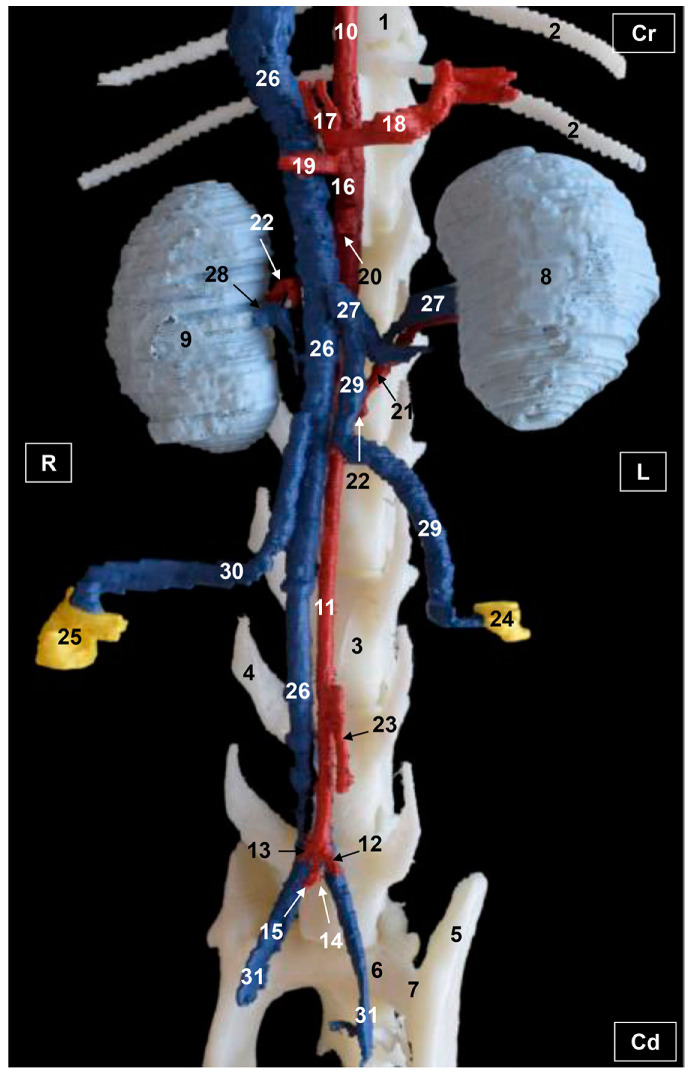
Three-dimensional reconstruction image of the abdominal and pelvic arterial and venous system in a female feline. 3D printing. Ventral view. R = Right. L = Left. Cr = Cranial. Cd = Caudal. 1. Thoracic vertebra; 2. ribs; 3. lumbar vertebra; 4. costiform process; 5. ilium bone: wing; 6. sacrum: ventral face; 7. sacroiliac joint; 8. left kidney; 9. right kidney; 10. descending aorta: thoracic aorta; 11. abdominal aorta; 12. left external iliac artery; 13. right external iliac artery; 14. left internal iliac artery; 15. right internal iliac artery; 16. celiac artery; 17. hepatic artery; 18. splenic artery; 19. left gastric artery; 20. cranial mesenteric artery; 21. left renal artery; 22. right renal artery; 23. caudal mesenteric artery; 24. left ovary; 25. right ovary; 26. caudal vena cava; 27. left renal vein; 28. right renal vein; 29. left ovarian vein; 30. right ovarian vein; and 31. left and right common iliac veins.

The visceral branches of the abdominal aorta, notably the celiac artery and its branches, as well as the cranial mesenteric artery were well visualized in 3D printing. However, these vascular structures were not fully appreciated in volumetric reconstructions. The adrenal and renal arteries were not observed with RadiAnt. With Amira, we were able to observe only the left renal artery. Both renal arteries were entirely identified with the OsiriX program and 3D printing. The ovarian arteries were not visible with any of the software used. With RadiAnt, we observed the entirety of the left uterine artery which arose from the vaginal artery, but the right uterine artery was not visible. The caudal mesenteric artery was seen but poorly represented with RadiAnt and Amira, whereas it was entirely identified with both OsiriX and 3D printing. Finally, the termination of the abdominal aorta was seen at the level of the joint between the sixth and seventh lumbar vertebrae, where the external and internal iliac arteries arose ventral to the end of caudal vena cava. The external iliac arteries were fully seen with Radiant, Amira and OsiriX but poorly represented with 3D printing. The internal iliac arteries were poorly represented with RadiaAnt and 3D printing but they were entirely observed with Amira and OsiriX. (Figure 5, Figure 6, Figure 7 and Figure 8).

#### 3.2.2. Venous System

The venous blood from the pelvic viscera and the pelvic limb drain into the internal and external iliac veins, which was seen with all the software used; while the uterine veins from the uterine horns and ovaries were observed but they were poorly represented with RadiAnt, Amira and OsiriX and not visible at all with 3D printing. Also, the uterine veins empty into the vaginal veins, which in turn drain into the internal iliac veins (right and left) before emptying into the common iliac veins (right and left) which are connected to the caudal vena cava. From the right ovary and right and left kidneys, blood drained directly into the caudal vena cava via the right ovarian vein and right and left renal veins. In addition, the left ovarian vein was entirely seen draining into the left renal vein. These details were observed with all the software used (Figure 5, Figure 6, Figure 7 and Figure 8).

Functional venous drainage from the intestines, stomach and spleen was observed through the splenic, cranial mesenteric and gastroduodenal veins that converged in the portal vein. The gastroduodenal vein was not visible at all on the images as it drains from the right side. However, the splenic, cranial mesenteric and portal veins were fully seen with RadiAnt and OsiriX but were not visible with Amira and 3D printing. In order to visualize the celiac and cranial mesenteric arteries, as well as the portal vein and the caudal vena cava the liver was partially removed. Finally, the right and left kidneys were observed in their entirety with all the software used (Figure 5, Figure 6, Figure 7 and Figure 8).

### 3.3. Magnetic Resonance Angiography

Four representative three-dimensional TOF MRA images of the abdominal and pelvic regions in different views were selected and presented. Thus, the TOF images are shown in several aspects (ventral, right and left oblique and right lateral). In the TOF images, we identified the liver, spleen, kidneys, ureters and ovaries, which allowed us to describe the principal arteries and veins, as well as their most important branches due to the high signal intensity.

#### 3.3.1. Arterial System

**Figure 9:**
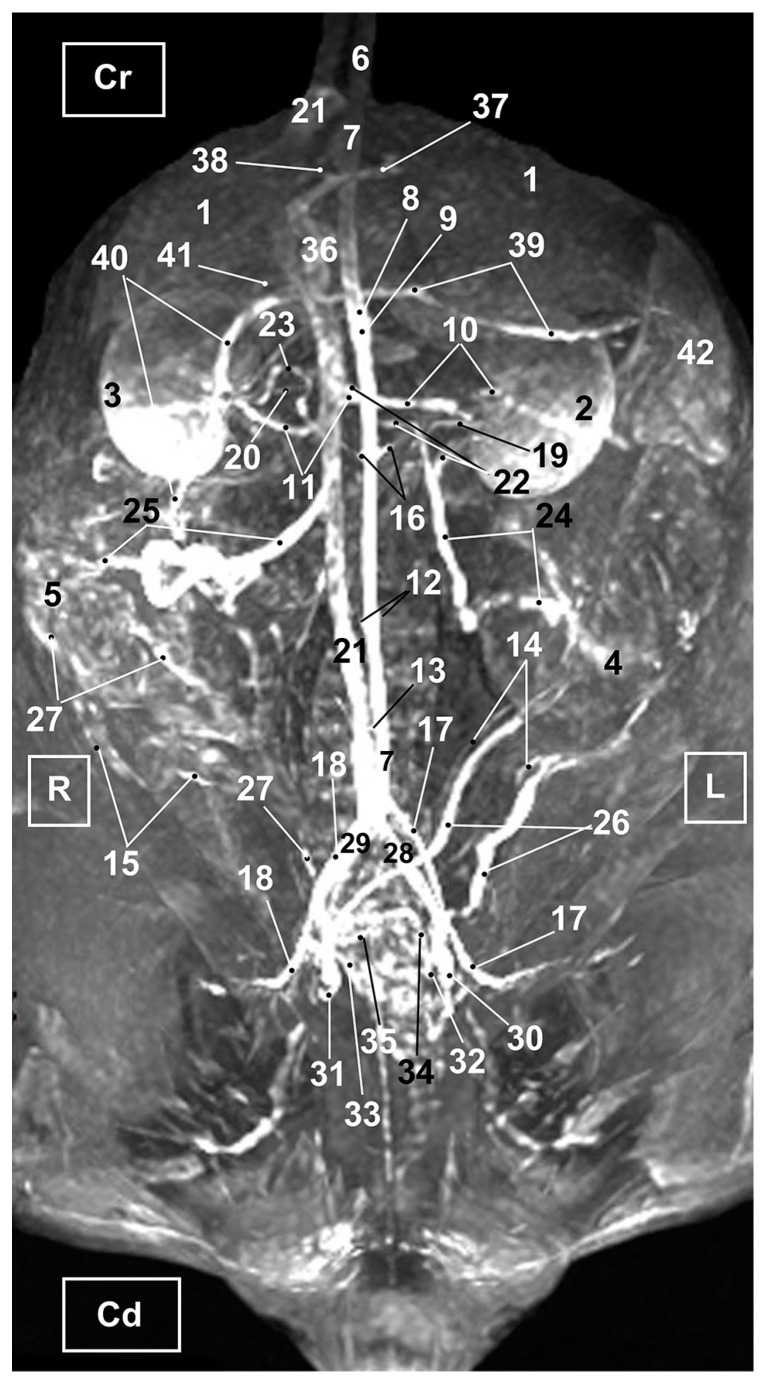
MRI TOF of the abdominal and pelvic arterial and venous system in the cat. Ventral view. R = Right side. L = Left side. Cr = Cranial. Cd = Caudal. 1. Liver; 2. left kidney; 3. right kidney; 4. left ovary; 5. right ovary 6. descending aorta: thoracic aorta; 7. abdominal aorta; 8. celiac artery; 9. cranial mesenteric artery. 10. left renal artery; 11. right renal artery; 12. right and left ovarian arteries; 13. caudal mesenteric artery; 14. left uterine artery; 15. right uterine artery; 16. right and left middle adrenal arteries; 17. left external iliac artery; 18. right external iliac artery; 19. left ureter; 20. right ureter; 21. caudal vena cava; 22. left renal vein; 23. right renal vein; 24. left ovarian vein; 25. right ovarian vein; 26. left uterine vein; 27. right uterine vein; 28. left common iliac vein; 29. right common iliac vein; 30. left external iliac vein; 31. right external iliac vein; 32. left internal iliac vein; 33. right internal iliac vein; 34. left vaginal vein; 35. right vaginal vein; 36. portal vein; 37. portal vein: left branch; 38. portal vein: right branch; 39. splenic vein; 40. cranial mesenteric vein; 41. gastroduodenal vein; and 42. spleen.

**Figure 10:**
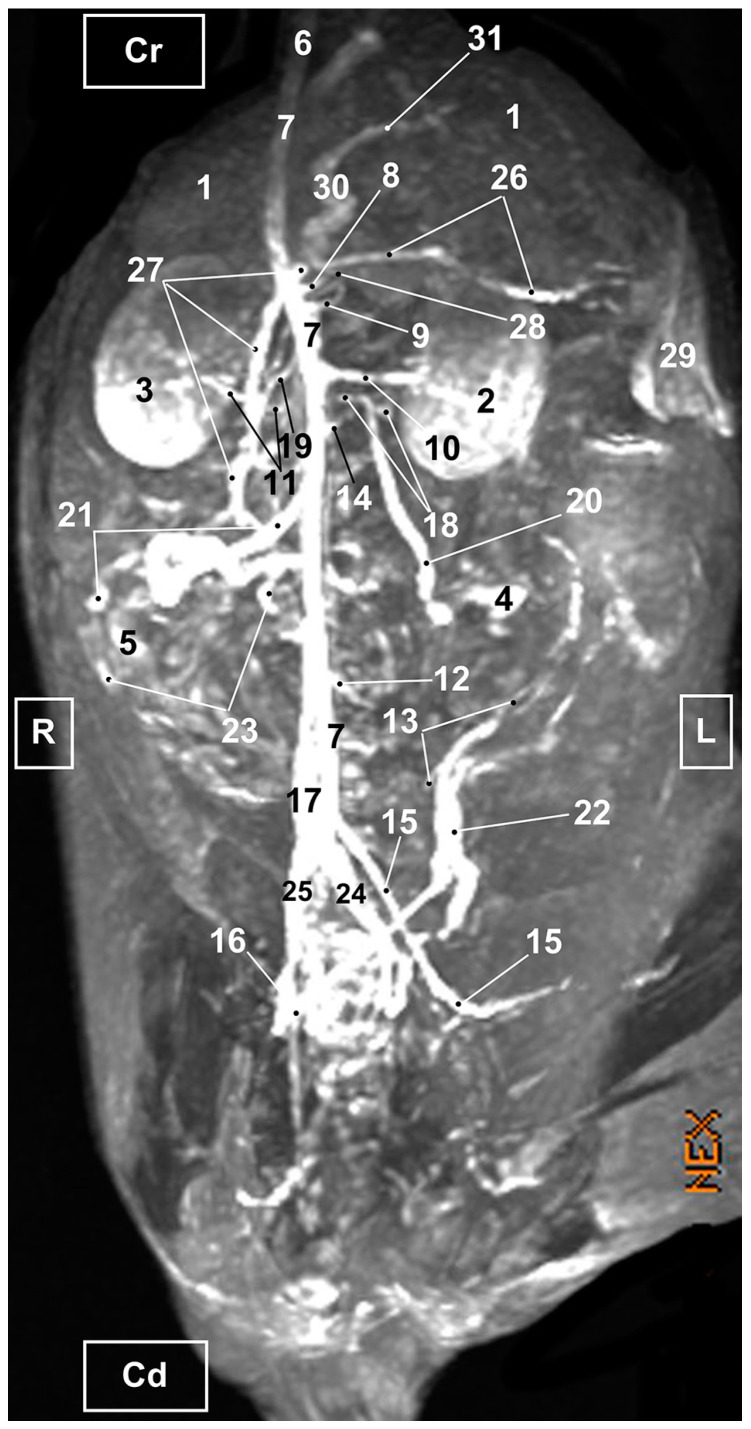
MRI TOF of the abdominal and pelvic arterial and venous system in the cat. Right oblique view. R = Right side. L = Left side. Cr = Cranial. Cd = Caudal. 1. Liver; 2. Left kidney; 3. Right kidney; 4. Left ovary; 5. Right ovary 6. Descending aorta: thoracic aorta; 7. Abdominal aorta; 8. Celiac artery; 9. Cranial mesenteric artery. 10. Left renal artery; 11. Right renal artery; 12. Caudal mesenteric artery; 13. Left uterine artery; 14. Right and left middle adrenal arteries; 15. Left external iliac artery; 16. Right external iliac artery; 17. Caudal vena cava; 18. Left renal vein; 19. Right renal vein; 20. Left ovarian vein; 21. Right ovarian vein; 22. Left uterine vein; 23. Right uterine vein; 24. Left common iliac vein; 25. Right common iliac vein; 26. Splenic vein; 27. Cranial mesenteric vein; 28. Gastroduodenal vein; 29. Spleen; 30. Portal vein; and 31. Portal vein: left branch.

**Figure 11:**
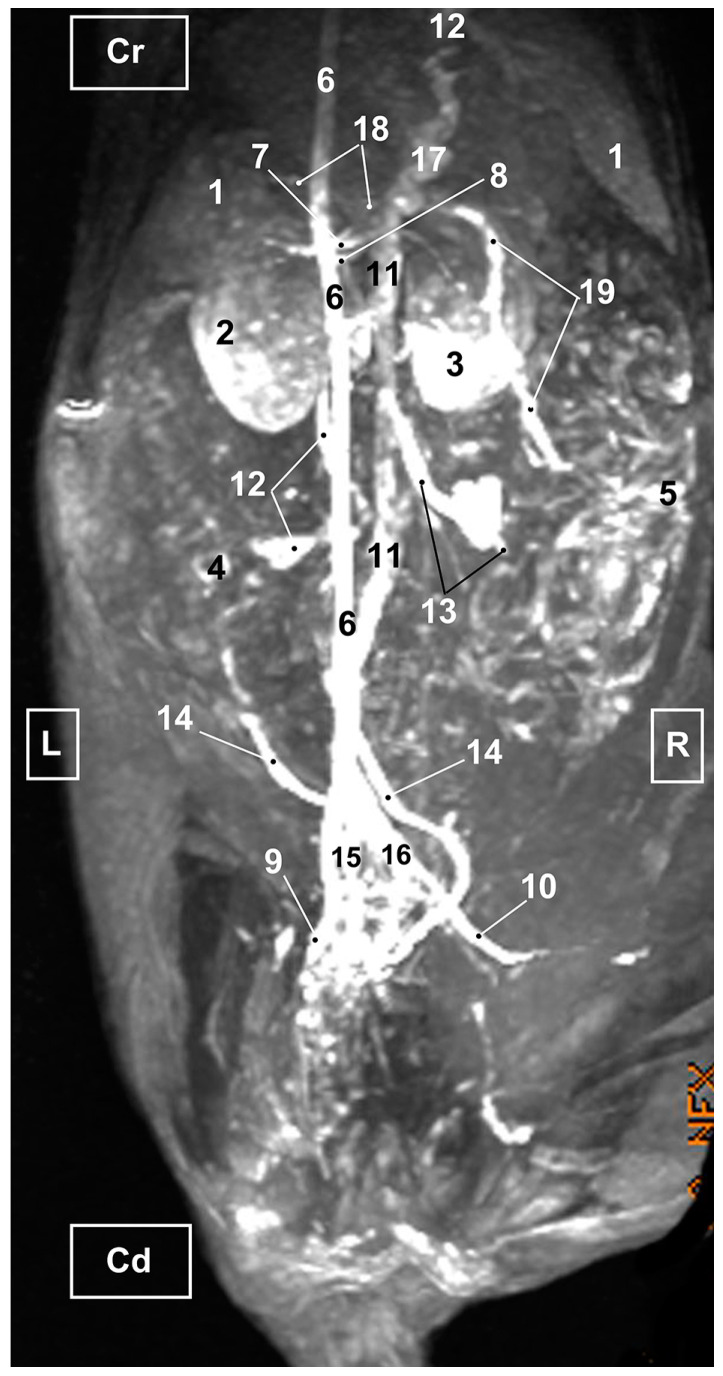
MRI TOF of the abdominal and pelvic arterial and venous system in the cat. Left oblique view. R = Right side. L = Left side. Cr = Cranial. Cd = Caudal. 1. Liver; 2. Left kidney; 3. Right kidney; 4. Left ovary; 5. Right ovary 6. Abdominal aorta; 7. Celiac artery; 8. Cranial mesenteric artery. 9. Left external iliac artery; 10. Right external iliac artery; 11. Caudal vena cava; 12. Left ovarian vein; 13. Right ovarian vein; 14. Left uterine vein; 15. Left common iliac vein; 16. Right common iliac vein; 17. Portal vein; 18. Splenic vein; 19. Cranial mesenteric vein.

**Figure 12:**
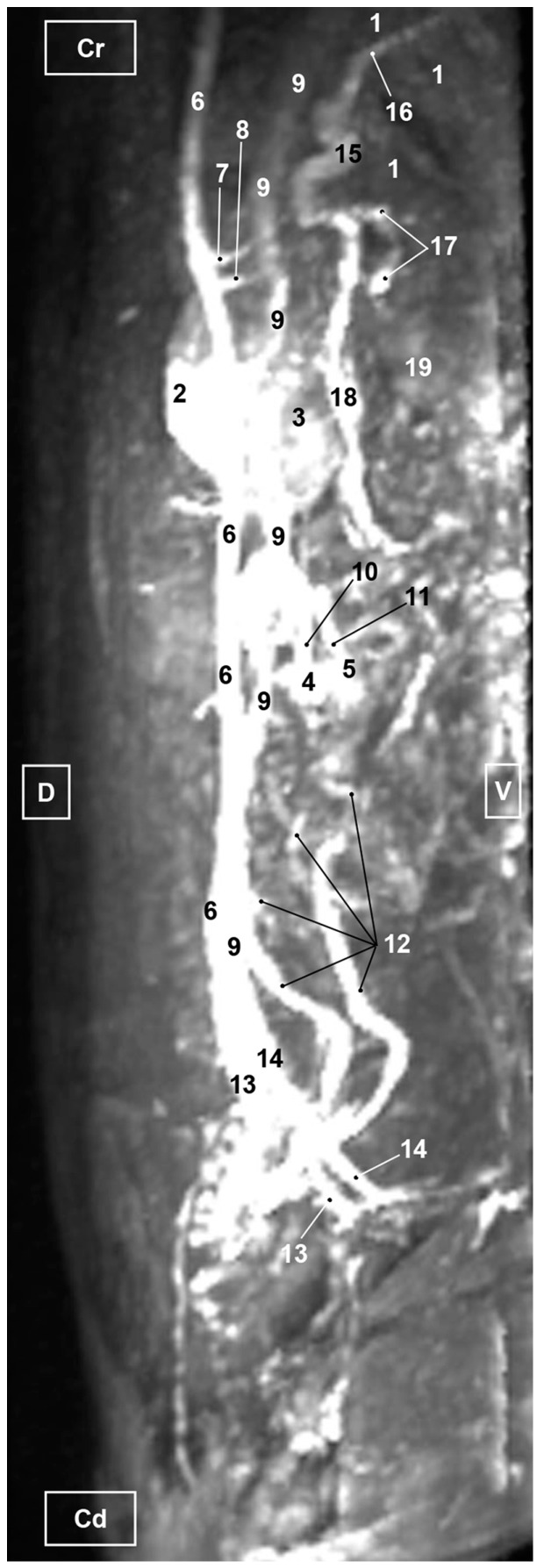
MRI TOF of the abdominal and pelvic arterial and venous system in the cat. Right lateral view. Cr=Cranial. Cd=Caudal. D=Dorsal. V=Ventral 1. Liver; 2. Left kidney; 3. Right kidney; 4. Left ovary; 5. Right ovary; 6. Abdominal aorta; 7. Celiac artery; 8. Cranial mesenteric artery; 9. Caudal vena cava; 10. Left ovarian vein; 11. Right ovarian vein; 12. Left uterine vein; 13. Left common iliac vein; 14. Right common iliac vein; 15. Portal vein; 16. Portal vein: right branch; 17. Splenic vein; 18. Cranial mesenteric vein; 19. Spleen.

The abdominal aorta, positioned to the left of the caudal vena cava was observed in its entirety. At the level of pelvic cavity, the bifurcation of the aorta into left and right external iliac arteries was clearly seen. Likewise, we identified the origins of the celiac, cranial mesenteric and ovarian arteries although these were not fully seen in the ventral view, whereas in the oblique and lateral views the vessels were seen but poorly represented. The other aortic branches (renal, caudal mesenteric, right uterine and right and left middle adrenal arteries) were all identified in the ventral (Figure 9) but not in the oblique (Figure 10 and Figure 11) or right lateral (Figure 12) views.

#### 3.3.2. Venous System

The TOF images showed the uterine veins flowing into the vaginal veins. These latter vessels joined the internal iliac veins, which in turn drained into the common iliac veins. Finally, they connected to the caudal vena cava (Figure 9, Figure 10, Figure 11 and Figure 12). In addition, the caudal vena cava was located to the right side of the aorta in the ventral view (Figure 9). In the right lateral view, the caudal vena cava was observed ventral to the abdominal aorta but poorly represented and the portal vein was identified but inadequately represented (Figure 12). The right ovarian vein can be seen in its entirety draining directly into the caudal vena cava and likewise the left ovarian vein flowing into the left renal vein (Figure 9 and Figure 10). Finally, the splenic, gastroduodenal and cranial mesenteric veins were accurately identified draining into the portal vein, which enters the liver at the hepatic porta, before dividing into right and left branches (Figure 9, Figure 10 and Figure 11).

## 4. Discussion

In the present study, feline abdominal and pelvic vascular structures were evaluated using gross dissections as an anatomical reference to compare with three-dimensional VR CTA as well as TOF images. Results from the current research show that the use of anatomical dissections injected with colored latex helps in the identification of the major blood vessels as well as their main branches and this enhances the anatomical accuracy of the study. Our study concurs with other authors who have used anatomical preparations with vascular repletion to facilitate the examination of the vasculature of the abdominal and pelvic cavities [25,27,28]. In our work, CTA images were acquired by means of a 16-slices spiral CT unit, which contributed a suitable view of the abdominal and pelvic vascular system. The reviewed literature contained a few studies on feline abdominal and pelvic anatomy using CTA [24,25,26,27,28,29]. Other researchers have applied CTA for the assessment of the characteristics of the renal vessels in healthy cats for the selection of appropriate feline renal transplant donors [30,31,32], and there is also one recent report that combine CTA VR reconstructions, anatomical dissections and 3D printings for the study of the vascularization and bile circulation of the feline liver [28]. Compared with previous works [24,25,26], we have tried to show 3D images of the branches of the great vessels (aorta and caudal vena cava) in the roof of the abdomen. Therefore, our study does not make a detailed examination of the vascularization of specific organs of the abdominal and pelvic cavity such as the liver [25,26,28] or kidneys [30,31,32].

For this study, the three-dimensional VR corresponding to CTA images of the abdomen and pelvis were made using 3D post-processing software: RadiAnt 2023.1, Amira 5.6 and OsiriX MD 13.0.2 DICOM Viewers. These images showed the main characteristics of the main arteries and veins as well as their regional branches. In all three-dimensional VR images, numerous arteries were well defined, including the thoracic and abdominal aorta, the external and internal iliac arteries, and the celiac artery. OsiriX MD 13.0.2 software provided the best views of the cranial and caudal mesenteric artery and renal arteries compared to RadiAnt VR in which we could only differentiate the cranial mesenteric artery; however, with the Amira reconstruction image we could observe both the cranial mesenteric and left renal arteries. Regarding the three software packages analyzed, we must state that VR is not available to veterinarians, sometimes, due to limited knowledge of the range of available software tools (Amira 5.6, OsiriX MD 13.0.2, RadiAnt 2023.1) [16]. An analytical study ranked OsiriX MD 13.0.2 and RadiAnt 2023.1 as the best software for performing VR [42]. In addition, the use of all reconstructed CTA acquisitions supplied accurate anatomical information on the main abdominal and pelvic veins.

In this study, three different software have been used, which are the ones that we can frequently find in the different areas of veterinary medicine. Thus, we chose OsiriX as it is one of the commercially available programs and is claimed to be the most widely used DICOM viewer in the world [43], especially among certified radiologists in veterinary referral centers and those doing teleradiology. In this project, OsiriX MD 13.0.2 software provided the best views of the vascular structures; however, we agree with [44] which highlights a disadvantage of OsiriX^®^ in that it is only compatible with the Mac^®^ operating system. The second software used was Amira 5.6, usually used in research institutions and universities. Several articles describe the use of this software [25,28,45,46,47,48]. In the present study, when comparing the use of Amira’s volumetric rendering image with that of OsiriX, it was not possible to observe certain veins as the portal and splenic veins or the internal and external iliac veins. Furthermore, it is an expensive software and is often not profitable outside large research centers or universities. The third software used in our study was RadiAnt 2023.1as it is considered an attractive package, applicable for all imaging modalities [49] and the preferred Windows DICOM viewer [42]. When comparing images obtained with RadiAnt with those of OsiriX, the internal and external iliac veins were not observed. However, RadiAnt showed the portal vein and its branches clearly, which were not seen with Amira.

For our study, a TOF bright-blood gradient-echo sequence was selected in ventral, right oblique, left oblique and right lateral view aspects. TOF images were very useful for identifying abdominal and pelvic vasculature. All vascular structures were identified based on high signal intensity compared to other tissues that showed less signal intensity. Thus, the image obtained in a ventral view allowed us to identify numerous arteries and veins in comparison with the right and left oblique, and right lateral aspects. This is due to the overlapping of structures when rotating the corresponding images.

In addition, the non-contrast TOF images allowed the identification of the course of the arteries and the veins, as well as their main ramifications. For better visualization of these abdominal and pelvic blood vessels, we have relied on the ventral aspect, and to a lesser extent, on the right and left oblique views, as the right lateral view did not assist in structure identification. In clinically healthy cats, similar non-contrast 3D TOF results were reported although these were compared with 3D ECG-FSE pulse sequence and contrast enhanced MRA. This study provided three-dimensional TOF images in dorsal and lateral views for the evaluation of the abdominal aorta and external iliac arteries [35]. However, our research of the abdominal and pelvic cavities used three-dimensional TOF images to observe accurately the main arteries and veins and their branches associated with the abdominal aorta and the caudal vena cava.

In small animals, anomalies of the portal system have been increasingly detected in recent years with the growing availability of advanced imaging techniques in veterinary practice [50,51,52]. Other congenital anomalies of the venous system using CTA have been reported [53,54,55]. Knowledge of the congenital and acquired anomalies and their consequences is of great importance for clinical decision-making in surgery and for planning interventional procedures [50,51,56].

In our study, three-dimensional CTA and MRA images of the abdominal and pelvic cavities provided accurate anatomical information of the main veins and would be useful in the evaluation of anomalous portal connections. The use of VR CT with contrast for detection of the extrahepatic segments of the portal vein (classic shunts: portocaval and its branches) is the most appropriate method for locating the anomaly and for planning surgery. The use of the RadiAnt viewer allowed highly detailed observation of the extrahepatic portal branches and other vessels (renal and ovarian). OsiriX provided an incomplete view, and observations were not possible with Amira. It is important to indicate that the ventral, oblique and lateral views should be used. The intrahepatic segment of the portal vein was completely observed using RadiAnt and OsiriX VR CT, although it took more time to remove adjacent tissues to fully appreciate it, whereas it was not completely observed with Amira. However, with MRA the oblique and lateral views showed the entirety of these segments although they were poorly represented in the ventral view.

Finally, 3D printing from cadaver cats was used in this study to facilitate a better interpretation of the CTA and the TOF images. In veterinary medicine, several reports of 3D printing and advanced corrosion casting technologies have supplied a valuable tool to aid surgery scheduling, improve anatomical knowledge and encourage research [25,27,28,36,37]. Colored latex vascular anatomical dissections, CTA acquisitions, TOF MRA and 3D printing of the feline abdominal and pelvic cavities have allowed us to identify the great vessels and their main branches. The correlation between RadiAnt, Amira and OsiriX images and 3D printing, and the vascularization of the feline abdominal and pelvic cavities could contribute to increasing knowledge of vascular anatomy among veterinary clinicians and researchers.

The main limitations of our study have been the number of healthy cats used (n = 2). It would be advisable to carry out more studies to confirm the results obtained. We decided to perform the CT study with a slice thickness of 3 mm as it is the standard thickness usually chosen in veterinary centers using a CT device, both in those that can opt for machines of only two detectors, and in those with more modern devices (16 or more detectors). We did obtain and analyze the arterial phase although it is not shown in this report; these arteries were observed in the venous phase that was used for the 3D reconstructions (VR).

## 5. Conclusions

The use of anatomical dissections with colored latex injections has allowed an accurate description of the vascular structures in CTA and MRA images. The images obtained through this study provided a basic anatomic reference aid to clinicians for the diagnosis of diseases of these regions and for further use and development of these techniques in feline medicine.

## Data Availability

The information is available at cuatrogatosadopciones@gmail.com; clinicasauces@clinicasauces.es.
